# Microstructural diffusion MRI for differentiation of breast tumors and prediction of prognostic factors in breast cancer

**DOI:** 10.3389/fonc.2025.1498691

**Published:** 2025-03-05

**Authors:** Xiaoyan Wang, Yan Zhang, Jingliang Cheng, Liangjie Lin, Ying Hu, Anfei Wang, Yong Zhang, Ruhua Wang, Ying Li, Kun Zhang, Wenhua Zhang

**Affiliations:** ^1^ Department of Magnetic Resonance Imaging, The First Affiliated Hospital of Zhengzhou University, Zhengzhou, China; ^2^ Clinical and Technical Support, Philips Healthcare, Beijing, China

**Keywords:** microstructural diffusion MRI, breast tumor, benign and malignant, molecular prognostic biomarker, IMPULSED method

## Abstract

**Purpose:**

This study aims to investigate the feasibility of cellular microstructural mapping by the diffusion MRI (IMPULSED, imaging microstructural parameters using limited spectrally edited diffusion) of breast tumors, and further to evaluate whether the MRI-derived microstructural features is associated with the prognostic factors in breast cancer.

**Materials and methods:**

This prospective study collected 232 patients with suspected breast tumors from March to August 2023. The IMPULSED MRI scan included acquisitions of diffusion MRI using both pulsed (PGSE) and oscillating (OGSE) gradient spin echo with the oscillating frequencies up to 33 Hz. The OGSE and PGSE data were fitted by the IMPUSLED method using a two-compartment model to estimate mean cell diameter (*d*_mean_), intracellular fraction (*f_in_*), extracellular diffusivity (*D*_ex_), and cellularity index (*f*_in_/d) within breast tumor lesions. The apparent diffusion coefficients (ADCs) were calculated from the conventional diffusion weighted imaging, PGSE, and OGSE (17 Hz and 33 Hz) sequences (ADC_DWI_, ADC_PGSE_, ADC_17Hz_, and ADC_33Hz_). The independent samples test was used to compare the *d*_mean_, *f_in_*, *D_ex_*, cellularity index, and ADC values between benign and malignant breast tumors, and between breast cancer subgroups with different risk factors. The receiver operating characteristic (ROC) curve was used to access the diagnostic performance.

**Results:**

213 patients were finally included and divided into malignant (n=130) and benign (n=83) groups according to the histopathological results. The *d*_mean_ (15.74 ± 2.68 *vs*. 14.28 ± 4.65 μm, p<0.001), *f*_in_ (0.346 ± 0.125 *vs*. 0.279 ± 0.212, p<0.001) and cellularity index (21.19 ± 39.54 *vs*. 19.38 ± 14.87 ×10-3 um^-1^, p<0.005) values of malignant lesions were significantly higher than those of benign lesions, and the *D*_ex_ (2.119 ± 0.395 *vs*. 2.378 ± 0.332 um^2^/ms, p<0.001) and ADC_DWI_ (0.877 ± 0.148 *vs*. 1.453 ± 0.356 um^2^/ms, p<0.001) of malignant lesions were significantly lower than those of benign lesions. For differentiation between benign and malignant breast lesions, ADC_DWI_ showed the highest AUC of 0.951 with the sensitivity of 80.49% and specificity of 98.28%. The combination of *d*_mean_, *f*_in_, *D*_ex_, and cellularity for differentiation between benign and malignant breast lesions showed AUC of 0.787 (sensitivity = 70.73%, and specificity = 77.86%), and the combination of IMPULSED-derived parameters with ADCs by PGSE and OGSE further improve the AUC to 0.897 (sensitivity = 81.93%, and specificity = 81.54%). The *f*_in_ values of HER-2(+) tumors were significantly lower than those of HER-2(-) tumors (0.313 ± 0.100 *vs*. 0.371 ± 0.137, p=0.015), and the ADC_DWI,_ ADC_17Hz_ and ADC_33Hz_ values of HER-2(+) tumors were significantly higher than those of HER-2(-) tumors (ADC_DWI_: 0.929 ± 0.115 *vs*. 0.855 ± 0.197 um^2^/ms, p=0.023; ADC_17Hz_: 1.373 ± 0.306 *vs*. 1.242 ± 0.301 um^2^/s, p =0.025; ADC_33Hz_: 2.042 ± 0.545 *vs*. 1.811 ± 0.392 um^2^/s, p = 0.008). The *f*_in_ (0.377 ± 0.136 *vs*. 0.300 ± 0.917, p=0.001) and cellularity index (27.22 ± 12.02 *vs*. 21.66 ± 7.76 ×10^-3^ um^-1^, p=0.007) values of PR(+) tumors were significantly higher than those of PR(-) tumor. The ADC_17Hz_ values of PR(+) tumors were significantly lower than those of PR(-) tumors(1.227 ± 0.299 *vs*. 1.404 ± 0.294 um^2^/s, p =0.002).The ADC_17Hz_ and *D*_ex_ values of ER(+) tumors were significantly lower than those of ER(-) tumors (ADC_17Hz_: 1.258 ± 0.313 *vs*. 1.400 ± 0.273 um^2^/s, p = 0.029; *D*_ex_: 2.070 ± 0.405 *vs*. 2.281 ± 0.331 um^2^/ms, p=0.011). For differentiation between ER(+) and ER(-), the ADC_17Hz_ and *D*_ex_ showed AUCs of 0.643 (sensitivity = 76.67%, and specificity = 47.06%) and 0.646 (sensitivity = 80.0%, and specificity = 45.98%), and the combination of *D*_ex_ and ADC_17Hz_ showed AUCs of 0.663 (sensitivity =93.33%, specificity = 36.78%). For differentiation of PR(+) and PR(-), the ADC_17Hz_, *f*_in_, and cellularity index showed AUCs of 0.666 (sensitivity = 68.18%, and specificity = 61.97%), 0.697 (sensitivity = 77.27%, and specificity = 60.27%) and 0.661 (sensitivity = 68.18%, and specificity = 61.64%), respectively, and their combination showed AUCs of 0.729 (sensitivity =72.73%, specificity = 65.75%). For differentiation of HER-2(+) and HER-2(-), the ADC_DWI_, ADC_17Hz_, and ADC_33Hz_, and *f*_in_ showed AUCs of 0.625 (sensitivity = 59.42%, specificity = 63.04%), 0.632 (sensitivity = 43.66%, and specificity = 84.78%), 0.664 (sensitivity = 47.95%, and specificity = 82.67%) and 0.650 (sensitivity = 77.46%, and specificity = 56.52%), respectively, and their combination showed AUCs of 0.693 (sensitivity = 69.57%, specificity = 64.79%) of HER-2(+) and HER-2(-).

**Conclusion:**

The IMPULSED method demonstrates promise for characterizing cellular microstructural features in breast tumors, which may be helpful for prognostic risk evaluation in breast cancer.

## 1 Introduction

In China, no matter in urban or rural areas, breast cancer ranks first in the spectrum of female cancer incidence and top 4 in the spectrum of female cancer death, and is also the most common type of cancer after lung cancer (1). Breast cancer is associated with complex biological behavior, and the classification of molecular subtypes can provide a basis for the formulation of treatment strategies and prognosis assessment for breast cancer patients (2). Perou et al. (3) proposed that expression of estrogen receptor (ER), progesterone receptor (PR), human epidermal growth factor receptor-2 (HER-2) and antigen identified by monoclonal antibody Ki-67 were the main factors determining the classification of breast cancer, which would guide the strategies for targeted therapy, endocrine therapy, or chemotherapy (4, 5). And efforts to identify molecular subtypes or prognostic factors of breast cancer using preoperative imaging have been ongoing.

Magnetic resonance imaging (MRI) is a non-invasive technique with exceptional soft tissue contrast and can provide anatomical and functional information on both normal and diseased tissues, such as tumors. MRI plays an important role in the diagnosis, treatment and prognosis assessment of breast diseases (6–8). However, traditional MRI can only reflect macroscopic features of a lesion, such as lesion size and morphology (9). Dynamic contrast-enhanced MRI (DCE-MRI) and diffusion-weighted imaging (DWI) based imaging biomarkers have been shown to be highly correlated with molecular subtypes and other prognostic and predictive factors in breast cancer (10). For DCE-MRI, due to the enhancement of background parenchyma and partial overlap of the time-intensity curves of benign and malignant lesions, the diagnosis by DCE-MRI is neither specific nor consistent (11). The conventional diffusion-weighted imaging (DWI) along with the derived apparent diffusion coefficient (ADC) has shown important diagnostic value in breast cancer, e.g., for discriminating malignancy. However, there is currently still no uniform standard of using ADC values for predicting the status of different tumor characteristic receptors (12). One of the key reasons may lie in that ADC is a general measurement of restricted diffusion rate that cannot pinpoint the underlying pathology; e.g., the change of cell size, cell density, and intra- or extra-cellular diffusivity (13).

The recently developed microstructural diffusion MRI methods, which captures the restriction of water diffusion at different diffusion length scales by varying diffusion times (td) and b values, have shown unique advantages in delineating cellular microstructures (14–16). In addition to the commonly used pulse gradient spin-echo (PGSE) sequence, which only allows td measurement on the order of tens of milliseconds on most clinical MRI scanners, the oscillating gradient spin-echo (OGSE) technique (15, 17) was usually introduced by microstructural diffusion MRI to achieve shorter td for probing microstructures at smaller scales. By incorporating the microstructural diffusion MRI with specific biophysical models, we can estimate important microstructural properties such as cell size, cell volume fraction, and transcytolemmal water exchange, which are closely related to the pathological changes of tumor (18). Among them, the IMPULSED (imaging microstructural parameters using limited spectrally edited diffusion) method (19) has been comprehensively validated using computer simulations in silico, cells *in vitro*, and animals *in vivo*. The MRI data acquisition for the IMPULSED method has also been successfully implemented in patients with breast (20) and prostate cancer (21–23) within clinically feasible scan times (eg, <7 minutes for breast imaging). Changes in cell sizes are typical features for both mitotic arrest (cell swelling) and apoptosis (cell shrinkage), for example, a cell in an early apoptotic stage may have a smaller diameter than a normal cell (22), therefore, measurements of cellular microstructures including cell size may provide a unique means for characterization of breast tumors associated with different kinds of risk factors.

The current study aims to evaluate the efficacy of microstructural mapping by the IMPULSED method in breast tumors, and further to evaluate whether the MRI-derived microstructural properties are associated with and can be used to predict the prognostic factors of breast cancer.

## 2 Methods

### 2.1 Patient characteristics

**Figure 1:**
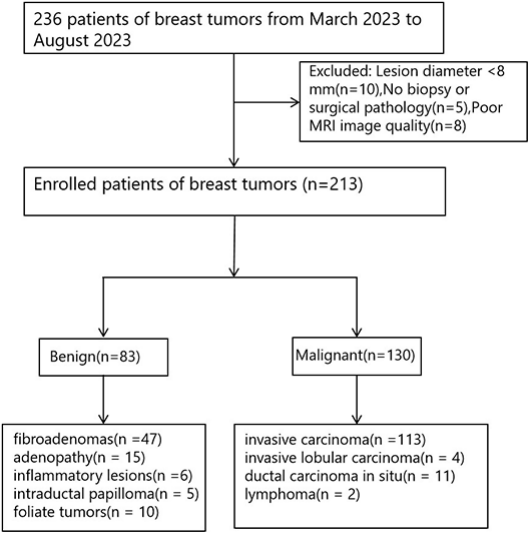
Flowchart shows participant enrollment.

This is a prospective study, and all participants were approved by our Clinical Research Ethics Review Committee. A total of 236 patients with clinical diagnosis of breast tumors from March 2023 to August 2023 were collected for breast MRI imaging. Inclusion criteria: 1) Suspicious breast lesions detected by mammography and/or ultrasound examination; 2) Patients who did not undergo puncture, radiotherapy or chemotherapy before MRI examination; 3) No MRI contraindications. Exclusion criteria: 1) Lesion diameter <8 mm (10 cases, small lesions will reduce the reliability of signal measurement); 2) No clear pathological or immunohistochemical results obtained after MRI scans (5 cases); 3) poor MRI image quality (8 cases). All enrolled patients were excluded due to one single exclusion criterion. Finally, 213 cases were enrolled, and the participant flowchart is shown in **Figure 1**.

### 2.2 MRI data acquisition

MRI was performed on a 3-T scanner (Ingenia Elition, Philips Healthcare, Best, the Netherlands) with the maximum gradient amplitude of 45 mT/m per axis, the maximum gradient slew rate of 220 mT/m/ms and a 7-channel breast coil. Routine pre-contrast MRI included T1WI, fat-suppressed T2WI, and conventional DWI. The DCE-MRI was used for anatomical reference. The IMPULSED MRI scan included acquisitions of diffusion MRI with both oscillating (OGSE) and pulsed (PGSE) gradient encoding using the oscillating frequencies up to 33 Hz. **Table 1** shows detailed parameters for all scans.

**Table 1:** Scan parameters for microstructural diffusion MRI.

	DWI	T1WI	T2WI	DCE-MRI	PGSE	OGSE_17Hz_	OGSE_33Hz_
TR (ms)	5480	541	4256.7	5.1	4000	4000	4000
TE (ms)	59.5	8	70	2.2	145	145	145
Field of views (mm^2^)	230×327	240×384	240×384	240×384	192×192	192×192	192×192
Voxel size (mm^3^)	2.8×3.3×4	1.0×1.2×4	1.0×1.11×4	1.00×1.0×1.60	2.53×2.58×5	2.53×2.58×5	2.53×2.58×5
Flip angle (°)	90	90	90	10	90	90	90
Matrix size	76×74×7	356×201×40	308×174×40	240×384×258	76×74×7	76×74×7	76×74×7
Reconstructed voxel size (mm^2^)	0.97×0.97×4	0.6×0.6×4	0.48×0.48×4	0.6×0.6×0.8	1.2×1.2×5	1.2×1.2×5	1.2×1.2×5
Cycle	/	/	/	/	/	1	2
f (Hz)	/	/	/	/	/	17	33
Effective td (ms)	36.8	/	/	/	26.7	15	7.5
Delta	44	/	/	/	119.2	72.7	72.7
delta	18	/	/	/	15.9	64.2	64.2
b-value (s/mm^2^)	/	/	/	/	0/250/500/750/1000/1400/1800	0/250/500/750/1000	0/100/200/300
Bandwidth (pixel/Hz)	76.6	224	207.9	947.0	37.2	37.2	37.2
Scan duration	2min28s	1min54s	2min8s	7min12s	4min24s	4min12s	2min8s

### 2.3 MRI data analysis

The IMPULSED parameters, including the mean cell diameter (*d*_mean_), intracellular fraction (*f*_in_), extracellular diffusivity (*D*_ex_), and cellularity index (*f*_in_/*d*_mean_), were estimated using a two-compartment model, with the intracellular diffusivity (*D*_in_) fixed at 1.58 μm^2^/ms to ensure fitting stability according to the previous study (19). The parameters were constrained to 4<d_mean_<30 μm, 0<f_in_ <1, and 0<D_ex_ <3.5 μm^2^/ms based on physiologically relevant values. The fitting was performed using the least square curve fitting toolbox in MATLAB (Mathworks, Inc.) according to a previous study (20) with the code available at https://github.com/jzxu0622/mati. Additionally, the ADC values for DWI, PGSE, and OGSE sequences were fitted according to S/S_0_ = exp(−b×ADC) (13).

The regions-of-interest (ROIs) for breast tumors were manually delineated on the slice with the largest scale of the lesion with reference to the high b value DWI and DCE-MRI images by experienced radiologists (H.Y with 13 years of experience and W.X.Y with 10 years of experience), and necrotic area and/or surrounding tissues were carefully excluded. The fitted microstructural parameters were calculated in a voxel-wise manner and averaged within the tumor ROIs.

### 2.4 Histopathological information

Two pathologists (with 8 and 12 years of experience, respectively) independently analyzed the hematoxylin and eosin staining and immunohistochemical results of the lesion specimens. Breast tumors were first divided into malignant and benign groups according to pathological results, and all the pathological results were obtained by operation. Besides, immunohistological staining of breast tumor excisions or biopsies provides the following information: hormone receptor (ER and PR) status, HER-2 status, and Ki-67 index. The criteria for positive expression of ER or PR were as follows: ER or PR were positive in ≥10% of tumor cells (24). The criteria for HER-2 status were as follows: samples of + and - signals were negative, and samples of +++ signals were positive; samples with a ++ signal were further hybridized *in situ* (samples with gene amplification were positive and samples without gene amplification were negative) (25). The criteria of Ki-67 expression were as follows: high expression was defined as staining positive in ≥14% of tumor cells, and low expression was defined as staining positive in < 14% of tumor cells (26). The concept of molecular typing of breast cancer was first proposed by Perou et al. (3), and breast cancer was divided into four main molecular subtypes through clustering analysis of gene expression profiles: Luminal Type A (ER+ and/or PR+, HER-2-), Luminal Type B (ER+ and/or PR+, HER-2+), HER-2 overexpression type (ER- and PR-, HER-2+), TN (triple-negative) type (ER- and PR-, HER-2-). The grade of invasive breast cancer (IBC) was evaluated according to pathological criteria, among which grade I was highly differentiated tumors; grade II, moderately differentiated tumor; and grade III, poorly differentiated tumor.

### 2.5 Statistical analysis

Statistical analyses were performed using Graphpad prism software (version 8.0, GraphPad Software, Inc., San Diego, CA, USA). Data homogeneity of variance was evaluated by Levene test. All quantitative measurements are expressed as mean ± standard deviation. The intraclass correlation coefficient (ICC) was used to evaluate the intra-observer reliability regarding the measurements of ADCs and cellular microstructural parameters. The independent samples t test was used to compare the *d*_mean_, *f_in_*, *D_ex_*, cellularity index, and ADC values between benign and malignant breast tumors, between breast cancer with different histological grading, between breast cancer with positive and negative expression of ER, PR, and HER-2, and between breast cancer with high and low expression of Ki-67, respectively. The receiver operating characteristic (ROC) curve was used to access the diagnostic performance of different imaging parameters in differentiation between benign and malignant tumors, as well as in recognition of different breast cancer risk factors. Logistic regression analyses were used to identify independent factors and combination diagnosis. P < 0.05 indicated that the difference was statistically significant.

## 3 Results

### 3.1 Patient characteristics

213 patients (45.12 ± 12 years old) with 213 tumor lesions (83 benign and 130 malignant) were included in the final analysis. Basic demographic and clinical information of the patients are summarized in **Table 2**. Among the 130 malignant breast tumors, 117 cases were recognized as IBC. For the 117 cases of IBC, 87 out of 117 (74.36%) were identified ER-positive and 30/117 (25.64%) were negative, 73 out of 117 (62.39%) were identified PR-positive and 44/117 (37.61%) were negative, 46 out of 117 (39.32%) were identified HER-2-positive and 71/117 (60.68%) were negative, 104 out of 117 (88.89%) were identified high expression of Ki-67 and 13/117 (11.11%) were low expression of Ki-67. Among the IBC, there were 4 cases of grade I, 67 cases of grade II, and 46 cases of grade III.

**Table 2:** Participant information and tumor characteristics.

Characteristics	Number
Demographics No. of patients Age, mean ± standard deviation (years)	213 45.12 ± 12
Tumor size, (cm)	27 ± 22
Menstruation state Premenopausal women Postmenopausal women	115(53.99%) 98(46.01%)
Benign	Fibroadenomas (n =47) Adenopathy (n = 15) Inflammatory lesions (n =6) Intraductal papilloma (n = 5) Foliate tumors (n = 10)
Malignant	Invasive carcinoma (n =113) Invasive lobular carcinoma (n = 4) Ductal carcinoma *in situ* (n = 11) Lymphoma (n = 2)
Malignant lesion tissue type IBC (N %) Grade I Grade II Grade III	4 (3.42%) 67 (57.26%) 46 (39.32%)
Cancer subtype Luminal A Luminal B HER2 overexpression type TN	8 (6.84%) 71 (60.68%) 26 (22.22%) 12 (10.26%)
ER status Positive Negative	87 (74.36%) 30 (25.64%)
PR status Positive Negative	73 (62.39%) 44 (37.61%)
HER-2 status Positive Negative	46 (39.32%) 71 (60.68%)
Ki-67 status High expression Low expression	104 (88.89%) 13 (11.11%)

### 3.2 Differences in microstructural parameters by IMPULSED between benign and malignant breast lesions

**Figure 2:**
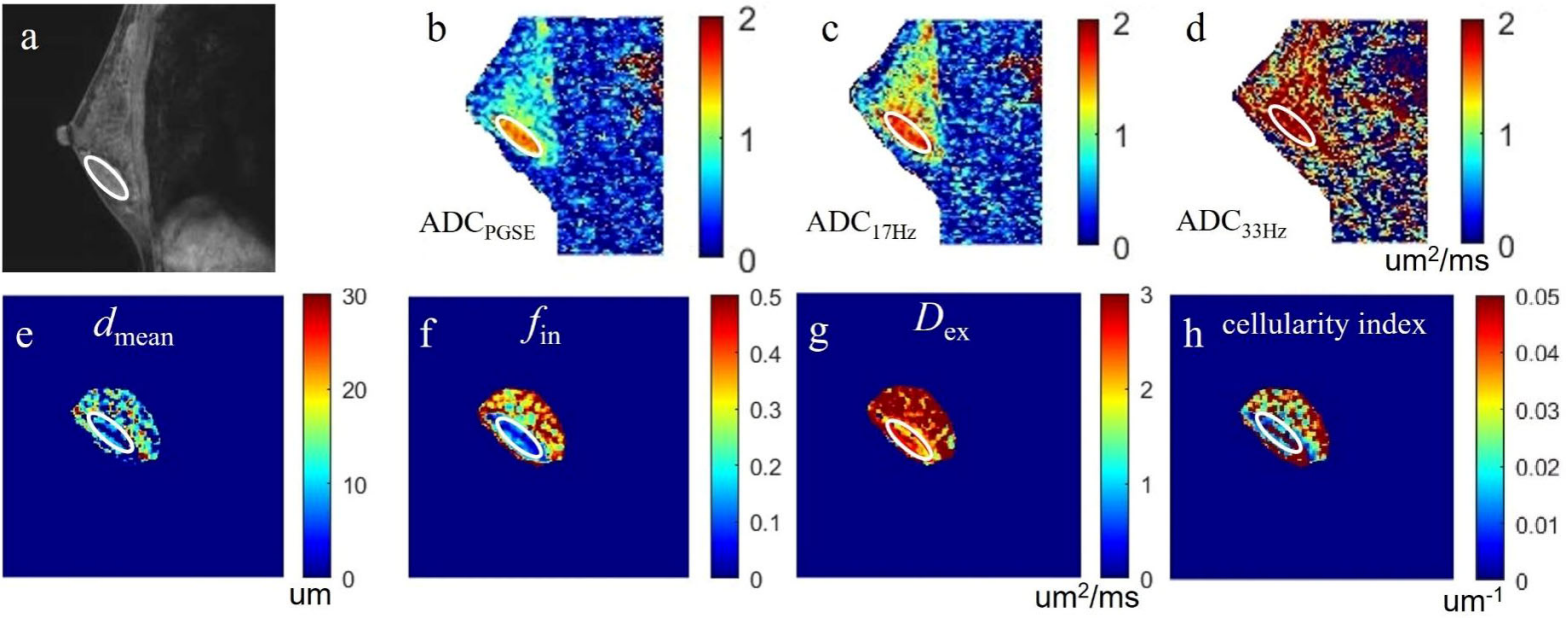
a case of fibroadenoma in the right breast: **(A)** the sagittal DCE-MRI image as reference; **(B-D)** the sagittal ADC_PGSE,_ ADC_17Hz_, and ADC_33Hz_ images with values for the lesion of 1.382, 1.589 and 2.523 um^2^/ms, respectively; **(E-H)** the *d*_mean_, *f*_in_, D_ex_, and cellularity index images around the lesion fitted by the IMPULSED method with values for the lesion of 10.38 um, 14.22%, 2.456 um^2^/ms, and 14.91×10^-3^ um^-1^, respectively. These microstructural parameters were only fitted at a limited region covering the lesion for saving of the post-processing time. The circles on the images are the ROIs of the lesion.

**Figure 3:**
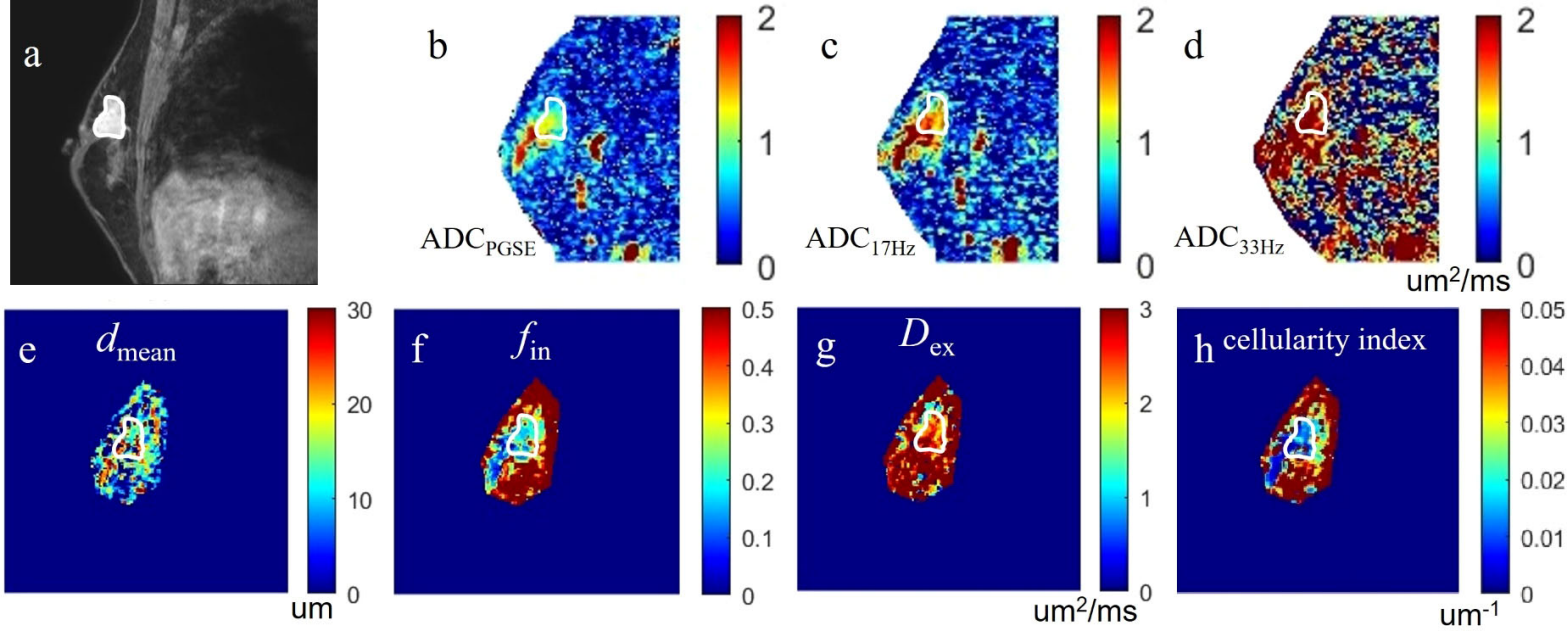
a case of invasive carcinoma in the right breast: **(A)** the DCE-MRI image as reference; **(B-D)** the ADC_PGSE,_ ADC_17Hz_ and ADC_33Hz_ images with values for the lesion of 1.021, 1.409, and 2.018 um^2^/ms, respectively; **(E-H)** the *d*_mean_, *f*_in_, D_ex_, and cellularity index images around the lesion fitted by the IMPULSED method with values for the lesion of 13.86 um, 25.53%, 2.267 um^2^/ms, and 21.24×10^-3^ um^-1^, respectively. These microstructural parameters were only fitted at a limited region covering the lesion for saving of the post-processing time. The circles on the images are the ROIs of the lesion.

The ICCs between the two observers for measurement of quantitative ADC and cellular microstructural parameters were all higher than 0.75, suggesting excellent reliability (**Table 3**). The microstructural parameters for benign and malignant tumors are shown in **Table 4**, and the representative images of patients in the two groups are shown in **Figures 2**, **3**. The *d*_mean_ *, f_in_* and cellularity index values of malignant lesions were significantly higher than those of benign lesions (15.74 ± 2.68 *vs*. 14.28 ± 4.65 μm, 0.346 ± 0.125 *vs*. 0.279 ± 0.212, 21.19 ± 39.54 *vs*. 19.38 ± 14.87 ×10^-3^ um^-1^, *d*_mean_ and cellularity p<0.001, *f*_in_ p<0.005), and the *D_ex_* and ADC values of malignant lesions were significantly lower than those of benign lesions (*D*_ex_: 2.119 ± 0.395 *vs*. 2.378 ± 0.332, ADC_DWI_: 0.877 ± 0.148 *vs*. 1.453 ± 0.356, ADC_PGSE_: 1.196 ± 0.379 *vs*. 0.853 ± 0.243, ADC_17Hz_: 1.582 ± 0.377 *vs*. 1.285 ± 0.468, and ADC_33Hz_: 2.180 ± 0.386 *vs*. 1.896 ± 0.473 um^2^/ms; all p<0.001). For both of benign and malignant breast lesions ADC_33Hz_ >ADC_17Hz_ > ADC_PGSE_ (benign: 2.180 ± 0.386 *vs*. 1.582 ± 0.377 *vs*. 1.196 ± 0.379 um^2^/ms, malignant: 1.896 ± 0.473 *vs*. 1.285 ± 0.468 *vs*. 0.853 ± 0.243 um^2^/ms). For differentiation between benign and malignant breast lesions, ADC_DWI_ showed the highest area under ROC curve (AUC, 0.951) (sensitivity = 80.49, and specificity = 98.28%). The ADC values by PGSE and OGSE sequences showed AUCs ranged from 0.728 to 0.753. The IMPULSED derived microstructural parameters, including *d*_mean_ *, f*_in_, *D*_ex_ and the cellularity index, showed the AUCs ranged from 0.630 to 0.700, and the diagnostic performance can be significantly improved with their combination (AUC = 0.787). The combination of IMPULSED-derived parameters and ADCs by PGSE and OGSE can further improve the AUC to 0.897 (sensitivity = 81.93%, and specificity = 81.54%).

**Table 3:** The interclass correlation coefficient and 95% confidence intervals for *d*_mean_, *f_in_*, *D_ex_*, cellularity index, and ADCs measurements between observers.

Parameters	Intraclass correlation coefficients (95% confidence intervals)
*d*_meam_	0.797(0.776-0.837)
*f_in_*	0.825(0.795-0.856)
*D*_ex_	0.815(0.781-0.847)
cellularity index	0.869(0.810-0.892)
ADC_DWI_	0.948(0.926-0.984)
ADC_PGSE_	0.869(0.815-0.892)
ADC_17Hz_	0.853(0.810-0.883)
ADC_33Hz_	0.846(0.803-0.873)

**Table 4:** Comparison of Microstructural diffusion MRI parameters between benign and malignant breast lesions, and between different subtypes or histological grades of breast cancer.

Parameters	*d*_meam_ (um)	*f*_in_	*D*_ex_ (um^2^/ms)	cellularity index (×10^-3^ um^-1^)	ADC_DWI_ (um^2^/ms)	ADC_PGSE_ (um^2^/ms)	ADC_17Hz_ (um^2^/ms)	ADC_33Hz_ (um^2^/ms)
Benign (n = 83)	14.38 ± 4.645	0.279 ± 0.212	2.378 ± 0.332	19.38 ± 14.87	1.453 ± 0.356	1.196 ± 0.379	1.582 ± 0.377	2.180 ± 0.386
Malignant (n = 130)	15.74 ± 2.677	0.346 ± 0.125	2.119 ± 0.395	21.19 ± 39.54	0.877 ± 0.148	0.853 ± 0.243	1.285 ± 0.468	1.896 ± 0.473
P	<0.001***	0.005**	<0.001***	0.001***	<0.001***	0.001***	0.001***	0.001***
ER(-) (n= 30)	15.71 ± 2.315	0.313 ± 0.117	2.281 ± 0.331	22.77 ± 10.05	0.902 ± 0.138	0.942 ± 0.235	1.400 ± 0.273	2.035 ± 0.416
ER(+) (n=87)	15.77 ± 2.797	0.360 ± 0.128	2.070 ± 0.405	25.94 ± 11.25	0.879 ± 0.185	1.028 ± 1.141	1.258 ± 0.313	1.855 ± 0.481
P	0.911	0.080	0.011*	0.171	0.529	0.684	0.029*	0.071
PR(-) (n=44)	15.90 ± 2.780	0.300 ± 0.917	2.183 ± 0.365	21.66 ± 7.76	0.899 ± 0.125	0.936 ± 0.229	1.404 ± 0.294	2.005 ± 0.552
PR(+) (n=73)	15.67 ± 2.621	0.377 ± 0.136	2.088 ± 0.413	27.22 ± 12.02	0.878 ± 0.198	1.048 ± 1.242	1.227 ± 0.299	1.839 ± 0.404
P	0.651	0.001***	0.210	0.007**	0.533	0.557	0.002**	0.064
HER-2(-) (n=71)	15.93 ± 2.807	0.371 ± 0.137	2.073 ± 0.415	26.31 ± 11.86	0.855 ± 0.197	1.063 ± 1.257	1.242 ± 0.301	1.811 ± 0.3921
HER-2(+) (n=46)	15.49 ± 2.456	0.313 ± 0.100	2.203 ± 0.357	23.31 ± 9.121	0.929 ± 0.115	0.918 ± 0.239	1.373 ± 0.306	2.042 ± 0.545
P	0.381	0.015*	0.083	0.149	0.023*	0.446	0.025*	0.008**
Ki-67(low) (n=13)	15.99 ± 2.267	0.384 ± 0.098	2.239 ± 0.433	26.89 ± 6.86	0.945 ± 0.136	0.788 ± 0.122	1.322 ± 0.279	2.063 ± 0.689
Ki-67(high) (n=104)	15.73 ± 2.727	0.345 ± 0.129	2.109 ± 0.392	24.91 ± 11.34	0.876 ± 0.177	1.033 ± 1.047	1.291 ± 0.313	1.881 ± 0.435
P	0.739	0.283	0.268	0.540	0.186	0.403	0.733	0.189
I-II (n=71)	16.11 ± 2.538	0.348 ± 0.123	2.082 ± 0.420	0.024 ± 0.010	0.889 ± 0.135	0.803 ± 0.117	1.161 ± 0.140	1.550 ± 0.314
III (n=46)	15.38 ± 2.520	0.340 ± 0.104	2.121 ± 0.331	0.025 ± 0.008	0.842 ± 0.128	0.845 ± 0.176	1.262 ± 0.236	1.879 ± 0.344
P	0.998	0.999	0.999	0.999	0.999	0.999	0.999	0.999

* represents p ≤ 0.05, ** represents p ≤ 0.01, and *** represents p ≤ 0.001.

### 3.3 Microstructural features for breast cancer with different immunophenotypes and pathological grades

**Figure 4:**
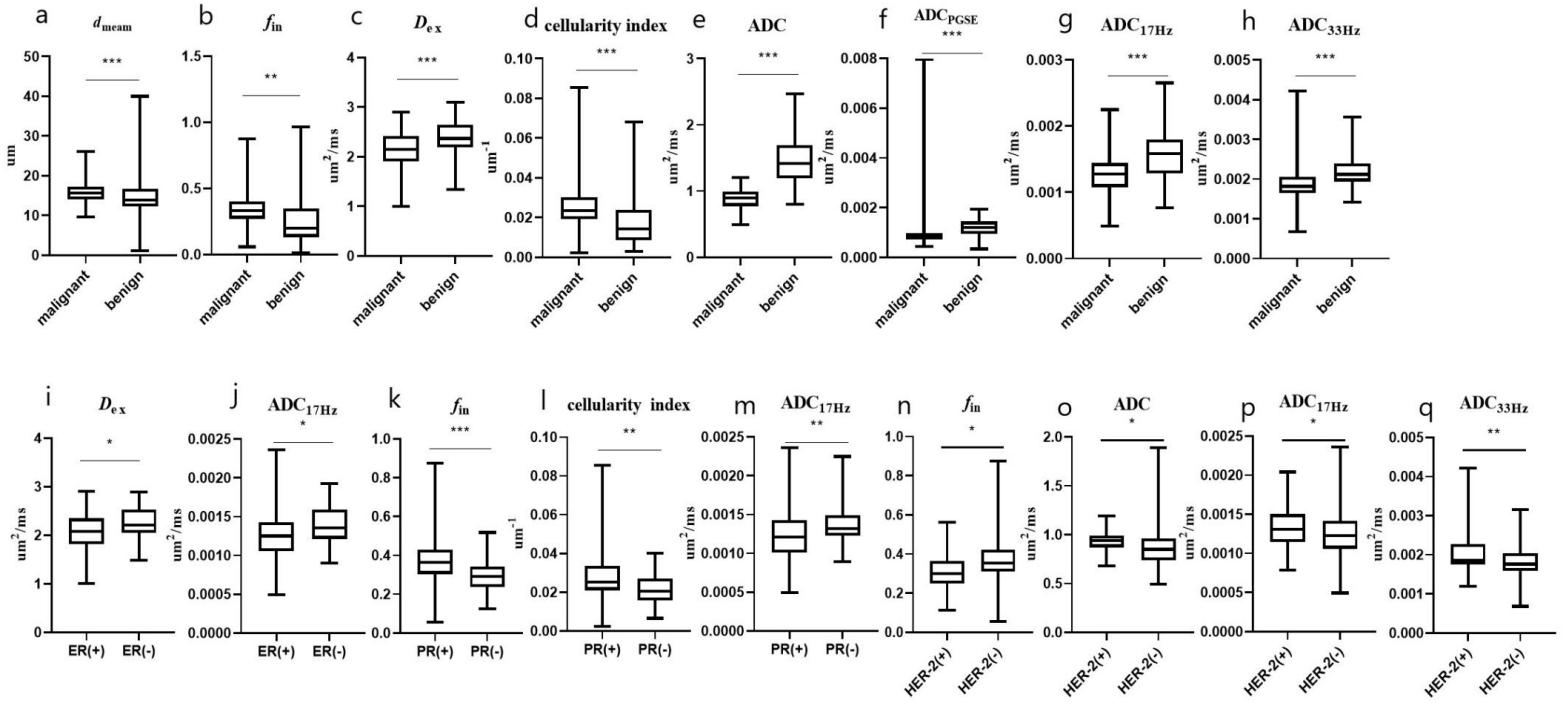
The microstructural parameters measured by the IMPUSED method with significant differences between benign and malignant tumors, or between different subtypes of breast cancer. The *d*_mean_ **(A)**, *f*_in_ **(B) **and cellularity index **(D)** of malignant lesions were significantly higher than those of benign lesions, and the *D*_ex_ **(C)** and ADCs **(E–H)** of malignant lesions were significantly lower than those of benign lesions; the *D*_ex_ and ADC_17Hz_ were lower in the ER(+) than in ER(-) group **(I, J)**; the *f*_in_ and cellularity index were higher in the PR(+) than in PR(-) group **(K, L)**; the ADC_17Hz_ values of PR(+) tumors were significantly lower than those of PR(-) tumors **(M)**; and the *f*_in_, ADC_DWI_, ADC_17Hz_ and ADC_33Hz_ values were higher in the HER-2(+) than in HER-2(-) group **(N–Q)**. * represents p ≤ 0.05, ** represents p ≤ 0.01, and *** represents p ≤ 0.001.

**Figure 5:**
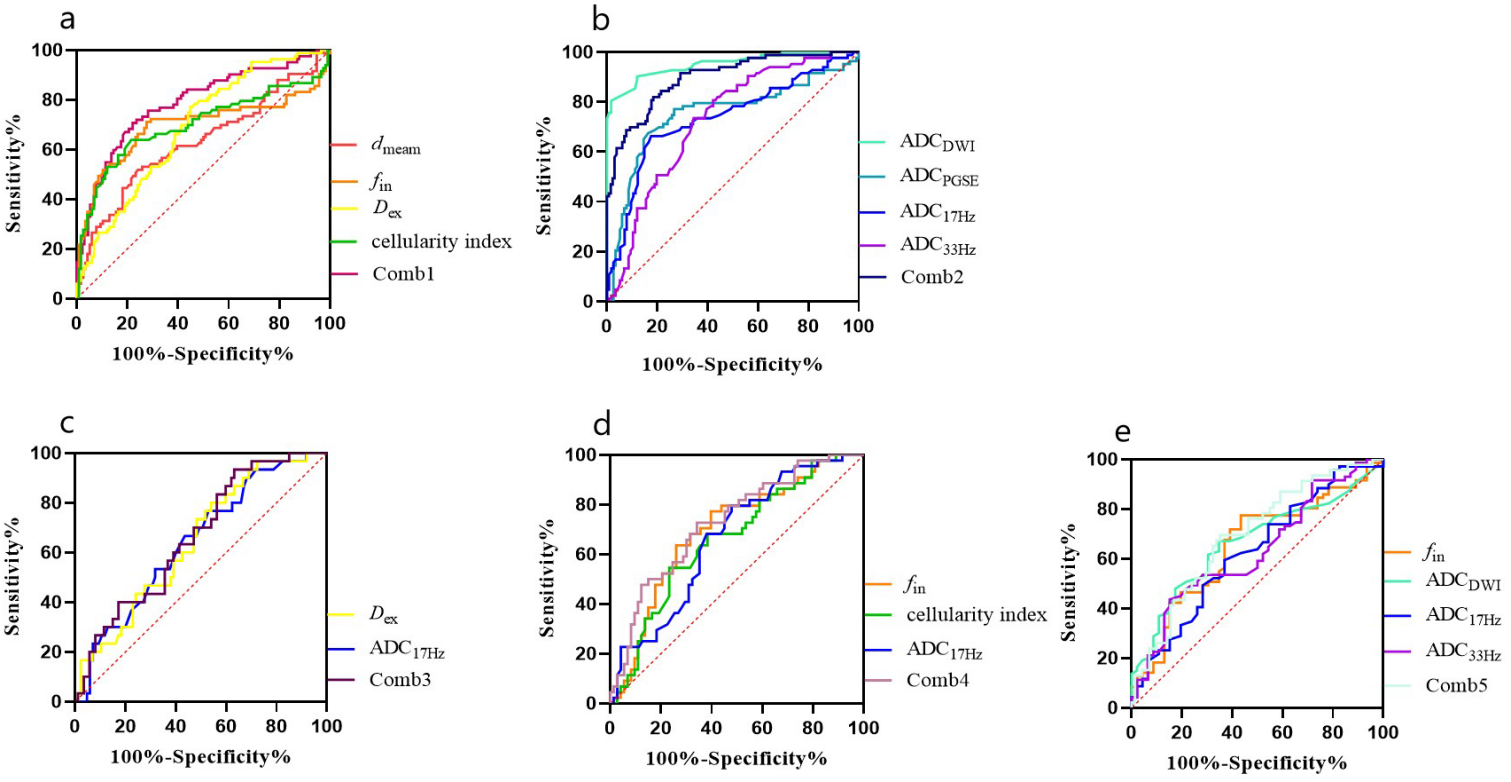
ROC curves for different parameters **(A, B)** for differentiation between malignant and benign breast lesions (Comb1: the combination of IMPULSED derived parameters, and Comb2: the combination of IMPULSED derived parameters and ADCs by PGSE and OGSE), and ROC curves of different parameters for differentiation between different subtypes of breast cancer (**C**: ER, **D**: PR, and **E**: HER-2).

The ADC_17Hz_ and *D_ex_* values of ER(+) tumors were significantly lower than those of ER(-) tumors (ADC_17Hz_: 1.258 ± 0.313 *vs*. 1.400 ± 0.273 mm^2^/s, p = 0.029; *D*_ex_: 2.070 ± 0.405 *vs*. 2.281 ± 0.331 um^2^/ms, p=0.011) (**Table 4**). The *f_in_* (0.377 ± 0.136 *vs*. 0.300 ± 0.917, p=0.001) and cellularity index (27.22 ± 12.02 *vs*. 21.66 ± 7.76 ×10^-3^ um^-1^, p=0.007) values of PR(+) tumors were significantly higher than those of PR(-) tumor. The ADC_17Hz_ values of PR(+) tumors were significantly lower than those of PR(-) tumors(1.227 ± 0.299 *vs*. 1.404 ± 0.294 mm^2^/s, p =0.002). The *f_in_* values of HER-2(+) tumors were significantly lower than those of HER-2(-) tumors (0.313 ± 0.100 *vs*. 0.371 ± 0.137, p=0.015), and the ADC_DWI_, ADC_17Hz_ and ADC_33Hz_ values of HER-2(+) tumors were significantly higher than those of HER-2(-) tumors (ADC_DWI_: 0.929 ± 0.115 *vs*. 0.855 ± 0.197 mm^2^/ms, p=0.023; ADC_17Hz_: 1.373 ± 0.306 *vs*. 1.242 ± 0.301, mm^2^/s, p =0.025; ADC_33Hz_: 2.042 ± 0.545 *vs*. 1.811 ± 0.392 mm^2^/s, p = 0.008). For differentiation between ER(+) and ER(-), the ADC_17Hz_ and *D_ex_* showed AUCs of 0.643 (sensitivity = 76.67%, and specificity = 47.06%) and 0.646 (sensitivity = 80.0%, and specificity = 45.98%), and the combination of *D*_ex_ and ADC_17Hz_ showed a AUC of 0.663 (sensitivity =93.33%, specificity = 36.78%). For differentiation of PR(+) and PR(-), the ADC_17Hz_, *f_in_*, and cellularity index showed AUCs of 0.666 (sensitivity = 68.18%, and specificity = 61.97%), 0.697 (sensitivity = 77.27%, and specificity = 60.27%) and 0.661 (sensitivity: 68.18%, and specificity: 61.64%), respectively, and their combination showed a AUC of 0.729 (sensitivity =72.73%, specificity = 65.75%). For differentiation of HER-2(+) and HER-2(-), the ADC_DWI_, ADC_17Hz_, and ADC_33Hz_, and *f_in_* showed AUCs of 0.625 (sensitivity = 59.42%, specificity = 63.04%), 0.632 (sensitivity = 43.66%, and specificity = 84.78%), 0.664 (sensitivity = 47.95%, and specificity = 82.67%) and 0.650 (sensitivity = 77.46%, and specificity = 56.52%), respectively, and their combination showed a AUC of 0.693 (sensitivity = 69.57%, specificity = 64.79%) (**Table 5**, **Figures 4**, **5**). There was no significant difference in the ADCs and quantitative microstructural parameters between breast cancer with low-to-moderate (I and II) and high (III) histological grade, as well as between breast tumors with high and low expression of Ki-67.

**Table 5:** Performance of the cellular microstructural parameters derived by IMPULSED in differentiation between benign and malignant breast lesions, as well as between different subtypes of breast cancer.

parameters	AUC	Sensitivity	Specificity	Cut-off value
benign *vs*. malignant				
d_meam_	0.630	51.81	75.86	14.02 um
*f_in_*	0.696	71.08	72.41	28.00%
D_ex_	0.688	77.11	55.17	2.180 um^2^/ms
cellularity index	0.700	63.86	78.45	18.95×10^-3^ um^-1^
Comb1	0.787	70.73	77.86	–
ADC_DWI_	0.951	80.49	98.28	1.115 um^2^/ms
ADC_PGSE_	0.753	67.47	83.62	1.035 um^2^/ms
ADC_17Hz_	0.737	66.27	82.46	1.475 um^2^/ms
ADC_33Hz_	0.728	73.49	65.52	1.995 um^2^/ms
Comb2	0.897	81.93	81.54	–
ER(+) *vs*. ER(-)				
D_ex_	0.646	80.00	45.98	2.033 um^2^/ms
ADC_17Hz_	0.643	76.67	47.06	1.223 um^2^/ms
Comb3	0.663	93.33	36.78	–
PR(+) *vs*. PR(-)				
*f_in_*	0.697	77.27	60.27	31.58%
cellularity index	0.661	68.18	61.64	24.65×10^-3^ um^-1^
ADC_17Hz_	0.666	68.18	61.97	1.215 um^2^/ms
Comb4	0.729	72.73	65.75	–
HER-2(+) *vs*. HER-2(-)				
*f_in_*	0.650	77.46	56.52	30.57%
ADC_DWI_	0.664	47.95	82.67	0.835 um^2^/ms
ADC_17Hz_	0.625	59.42	63.04	1.285 um^2^/ms
ADC_33Hz_	0.632	43.66	84.78	1.705 um^2^/ms
Comb5	0.693	69.57	64.79	–

Comb1 is the combination of *d*_mean_, *f*_in_, *D*_ex_, and cellularity index; Comb2 is the combination of IMPULSED and ADC; Comb3 is the combination of D_ex_ and ADC_17Hz_; Comb4 is the combination of *f*_in,_ cellularity index and ADC_17Hz_; Comb5 is the combination of *f*_in,_ADC_DWI,_ADC_17Hz_ and ADC_33Hz._

## 4 Discussion

In our study, we used the microstructural diffusion MRI (IMPULSED) to evaluate the microscopic characteristics of breast tumors and found that the microstructural parameters and ADC values showed significant differences between malignant and benign lesions. The microstructural parameters and/or ADC values also showed potential for non-invasive prediction of different prognostic risk factors in breast cancer.

Our results showed that the *d*_mean_ *, f_in_* and cellularity index values of malignant lesions were significantly higher than those of benign lesions, and the *D_ex_* and ADCs of malignant lesions were significantly lower than those of benign lesions, which is mostly in agreement with studies by Xu et al. (20) and Wu et al (21). Previous studies have shown that the ADC value is an effective parameter in differentiating benign and malignant breast lesions (27). Malignant tumors usually have lower ADC values than benign lesions due to their high cell density (28, 29), at the same time, the restriction of cell biofilm and the adsorption of macromolecules such as proteins on water molecules are also enhanced. The combined effect of these factors prevents the effective movement of water molecules in malignant tumors, thus reducing the ADC value, consistent with our findings. Lima et al. (30) found that ADC values of breast tumors increased with the shortening of diffusion time (increasing of gradient oscillation frequency) (ADC_PGSE_<ADC_OGSE_), and all the ADC values (by PGSE and OGSE) of malignant breast tumors were lower than those of benign breast tumors, which is consistent with our study. The combination (AUC = 0.897) of IMPULSED-derived parameters and ADCs by PGSE and OGSE show significant improvement in the diagnostic performance when compared to results by individual parameters (AUC = 0.630-0.753). Compared with benign lesions, the proliferation rate of malignant lesions was faster, the cell density was higher, and the extracellular space was reduced, which explained that *d*_mean_, *f*_in_ and cellularity index were higher in malignant lesions than in benign lesions. When distinguishing benign and malignant lesions, our results showed that ADC had the best performance among all the quantitative measurements, followed by the different microstructural features. Wu et al. (21) showed that the cellularity index had an AUC of 0.96 in distinguishing clinically significant from clinically insignificant prostate tumors, which is better than traditional ADC measurements. The lower AUCs of the IMPULSED derived microstructural parameters (compared to ADC) in our study may be related to the complex tissue composition of breast lesions. The advanced and complex model fitting of the IMPULSED method may also suffer from lower image quality and contribute to greater intra-group variation.

In breast cancer, the status of IHC (Immunohistochemistry) tumor receptors determines the subtype of breast cancer and is closely related to the cellular, vascular, and aggressive nature of the tumor (18). HER-2 is a transmembrane tyrosine kinase receptor, and its overexpression in breast cancer is a major factor in tumor progression and metastasis (31, 32). Her2-positive cells have a more malignant phenotype that stimulates excessive cell proliferation, invasion, and metastasis (33). Our research showed that the *f_in_* was significantly lower in the HER-2(+) group compared to its negative counterpart, while the ADC_DWI_, ADC_17Hz_ and ADC_33Hz_ were significantly higher, which is in line with the previous reports by Catalano et al (34). The positive expression of HER-2 may lead to increased microcirculation perfusion in tumor tissue, and the limited diffusion of water molecules in tissue and increased blood perfusion may jointly affect the ADC value of tumor, resulting in increased ADC value in HER-2 positive tumors. The lower *f_in_* was observed in HER-2(+) than in HER-2(-) tumors, which may be related to the increase of water exchange across the membrane in HER-2-overexpressing breast tumors. Previous studies (35) found that if transmembrane water exchange could not be ignored, the intracellular volume fraction would be essentially underestimated for any biophysical diffusion method that assumes no water exchange (including the IMPLUSE method). Besides, the reduced *f*_in_ may also indicate the more presence of necrotic core in HER-2(+) tumors, which is mainly composed of fluid and cell debris with reduced diffusion limitation (36).

ER and PR are hormone receptors that are known to be good prognostic factors, and in the presence of both receptors, treatment is effective for adjuvant or palliative hormone therapy. The cellularity index and *f_in_* were significantly higher in the PR(+) groups compared to their negative counterparts. This is consistent with the results of *f*_in_ increase in the PR(+) group in the previous study by BaR et al. (13). The cellularity index is calculated as the quotient of intracellular volume fraction and IMPULSED-derived cell diameter, and thus is proportional to the intracellular volume fraction. Our results also show that the ADC_17Hz_ value of PR(+) is lower than that of PR(-), which is basically consistent with the previous study by Ba et al. (13), which may be related to the differences in membrane permeability between different PR-expressing tumors. The *D_ex_* was significantly lower in the ER(+) group compared to its negative counterpart. The previous study has reported that ER-positive tumors were highly cellular (37). Animal model studies have also shown that angiogenic markers were inhibited when ER was overexpressed. All of these may result in the reduced *D*_ex_ values (38) in ER(+) tumors. The results of this study show that the ADC_17Hz_ value of ER(+) tumors is lower than that of ER(-) tumors, which may be due to the inhibitory effect of high level of ER expression on the angiogenic pathway of breast cancer (39).

Our study showed no statistical significance in quantitative microstructural diffusion MRI parameters and ADC values between low and high grade histological classification of malignant breast lesions, which is consistent with previous studies demonstrating no direct relationship between cell number and tumor grade (40, 41). Ki-67 index in tumor tissue is currently recognized as a marker of aggressive behavior in breast cancer. The microstructural diffusion MRI parameters and ADC values showed no significant difference between breast tumors with high and low expression of Ki-67. This is consistent with the results in the previous study by BaR, et al (13).

The current study has several limitations. First, although the consistency between IMPULSED-derived parameters and pathological results have been verified in previous studies (13, 20), it is still necessary for the current study to present such verification. However, the original pathological data for patients were unavailable to us, therefore, the related comparison was not presented in this study. Secondly, the number of some pathological type tumors was still relatively small, further investigation in a larger population is needed to verify the results of this study.

In conclusion, we have demonstrated the diagnostic potential of microstructural diffusion MRI based on the IMPULSED method for non-invasive exploration of cellular microstructural features in breast cancer in a clinical setting, and the feasibility of IMPULSED-derived parameters in differentiating breast cancer immunophenotypes. Results showed significant potential of microstructural diffusion MRI in discrimination of breast cancer immunophenotypes including the different expression status of ER, PR and HER-2.

## Data Availability

The raw data supporting the conclusions of this article will be made available by the authors without undue reservation.
